# Predictive value of the Naples prognostic score on postoperative delirium in the elderly with gastrointestinal tumors: a retrospective cohort study

**DOI:** 10.1186/s12877-024-05113-y

**Published:** 2024-06-20

**Authors:** Chenhao Song, Dongdong Yu, Yi Li, Meinv Liu, Huanhuan Zhang, Jinhua He, Jianli Li

**Affiliations:** Department of Anesthesiology, Hebei General Hospital, Shijiazhuang, Hebei Province 050051 China

**Keywords:** Naples prognostic score, Postoperative delirium, Gastrointestinal tumors, Elderly patients, Predictive markers

## Abstract

**Background:**

Postoperative delirium (POD) is a common complication among elderly patients after surgery. The Naples Prognostic Score (NPS), a novel prognostic marker based on immune-inflammatory and nutritional status, was widely used in the assessment of the prognosis of surgical patients. However, no study has evaluated the relationship between NPS and POD. The aim of this article was to investigate the association between NPS and POD and test the predictive efficacy of preoperative NPS for POD in elderly patients with gastrointestinal tumors.

**Materials and methods:**

In the present study, we retrospectively collected perioperative data of 176 patients (≥ 60 years) who underwent elective gastrointestinal tumor surgery from June 2022 to September 2023. POD was defined according to the chart-based method and the NPS was calculated for each patient. We compared all the demographics and laboratory data between POD and non-POD groups. Univariate and multivariate logistic regression analysis was used to explore risk factors of POD. Moreover, the accuracy of NPS in predicting POD was further assessed by utilizing receiver operating characteristic (ROC) curves.

**Results:**

20 had POD (11.4%) in a total of 176 patients, with a median age of 71 (65–76). The outcomes by univariate analysis pointed out that age, ASA status ≥ 3, creatinine, white blood cell count, fasting blood glucose (FBG), and NPS were associated with the risk of POD. Multivariate logistic regression analysis further showed that age, ASA grade ≥ 3, FBG and NPS were independent risk factors of POD. Additionally, the ROC curves revealed that NPS allowed better prognostic capacity for POD than other variables with the largest area under the curve (AUC) of 0.798, sensitivity of 0.800 and specificity of 0.667, respectively.

**Conclusion:**

Age, ASA grade ≥ 3, and FBG were independent risk factors for POD in the elderly underwent gastrointestinal tumor surgery. Notably, the preoperative NPS was a more effective tool in predicting the incidence of POD, but prospective trials were still needed to further validate our conclusion.

**Trial registration:**

The registration information for the experiment was shown below. (date: 3rd January 2024; number: ChiCTR2400079459)

**Supplementary Information:**

The online version contains supplementary material available at 10.1186/s12877-024-05113-y.

## Introduction

As the population of elderly individuals continues to grow, the number of older adults undergoing surgery is also increasing [1]. It has been reported that a considerable proportion of all surgical patients are undergoing surgery for gastrointestinal tumors, such as gastric cancer (GC) and colon cancer (CC) [2]. In the elderly population, the prevalence of postoperative complications is higher, leading to a poorer prognosis [1]. Postoperative delirium (POD), a familiar postoperative complication, occurred in 10.9–14.0% of the elderly who underwent gastrointestinal tumor surgery [3]. POD was often observed hours or days after surgery, which was mainly embodied in the rapid changes of attention, consciousness, cognitive function, perception, psychomotor activity and sleep pattern [4]. Moreover, POD was associated with an increased occurrence of other postoperative comorbidities and adverse outcomes, such as diminished quality of life, prolonged length of stay, aggravated medical burden, and even augmented in-hospital mortality [5]. Therefore, the exploration of valuable predictive factors was significant for preventing and mitigating POD in patients who underwent gastrointestinal tumors.

To date, several investigators proposed a multitude of potential blood indicators for predicting POD [5]. Specifically, several inflammatory parameters (e.g., IL-6, IL-8, S-100 β) in serum or cerebrospinal fluid (CSF) had been deemed to be associated with POD [6]. In addition, the preoperative monocyte-to-lymphocyte ratio (MLR) was a reliable predictor for POD in intensive care unit (ICU) patients underwent cardiac surgery [7]. Furthermore, recent literature showed a strong association between levels of nutritional parameters such as serum albumin (Alb) and lipids and an increased risk of POD [8]. However, it is important to note that the prognostic power of a single marker of inflammation or nutritional index might be influenced by variations in physical conditions and the surrounding environment [9]. Accordingly, the concept of using a compound indicator of nutrition state and inflammation, known as NPS, presented a novel idea for predicting POD. Additionally, it was reported that early rehabilitation, early discontinuation of analgesics, and reduced duration of mechanical ventilation could reduce the incidence of POD [10–12], therefore, clarifying the association between preoperative NPS and POD might help us to carry out appropriate preventive measures in advance in high-risk populations of POD.

The Naples prognostic score (NPS), originally proposed by Galizia et al., was composed of preoperative Alb, total cholesterol (TC), neutrophil-to-lymphocyte ratio (NLR), and lymphocyte-to-monocyte ratio (LMR), reflecting the inflammatory and nutritional immune status of the body [9]. Over the last few years, the NPS was widely used to evaluate the prognosis of cancer patients. Miyamoto et al. pointed out that the NPS was a valuable predictor for postoperative harmful outcomes in patients who underwent CRC and GC surgeries [13]. Another article affirmed that preoperative NPS served as a robust prognostic factor in patients with ampullary cancer, demonstrating superior prognostic performance compared to other individual nutritional or inflammatory biomarkers [14]. Besides, the available evidence suggested that preoperative NPS was independently related to the prevalence of postoperative syndromes [15]. Despite this, there has been limited research on the role of NPS in predicting POD for the elderly with gastrointestinal tumors. Briefly, such a correlation remained under-explored in NPS and POD for elderly patients who underwent surgery of gastrointestinal tumors.

Consequently, the aim of this retrospective trial was to identify NPS and other risk factors associated with delirium and to assess the predictive value of preoperative NPS for POD in the elderly underwent surgery for gastrointestinal tumors, which could identify high-risk populations of POD in advance and provide better guidance for the perioperative clinical care of patients.

## Materials and methods

### Ethics approval

This research was a single-center, retrospective cohort study and had been approved by the Medical Ethics Committee (approval No. 2023405) of the Hebei General Hospital. Since it was a retrospective study, we applied for and obtained consent for a waiver of informed consent from the medical ethics committee. Also, this article adhered to the Strengthening the Reporting of Observational Studies in Epidemiology (STROBE) guidelines. The study was successfully registered with the Chinese Clinical Trial Center (number: ChiCTR2400079459).

### Patient cohort

Clinical information on all patients who were hospitalized in the department of gastrointestinal surgery in Hebei General Hospital and underwent elective gastrointestinal tumor surgery was retrospectively collected from June 2022 to September 2023. Inclusion criteria: (i) the elderly age ≥ 60 years; (ii) American Society of Anesthesiologists (ASA) classification II∼IV; (iii) patients who underwent gastrointestinal tumor surgery under general anesthesia, including GC, rectal cancer (RC), and colon cancer (CC), etc. (iv) patients who had a complete blood sample were taken preoperatively. Exclusion criteria: (i) patients with a history of mild cognitive impairment (MCI), dementia, and delirium; (ii) patients with the duration of operation < 90 min or postoperative hospitalization < 3 days; (iii) patients with known preoperative infection or significant postoperative infections (pulmonary infection, urinary infection, and sepsis) occurred within 48 h after the operation. All the laboratory data were collected from the results of routine blood examinations within one week prior to surgery.

### Clinical information collection

The following baseline features, presurgical laboratory indicators and postoperative outcomes were extracted from the electronic medical record.

#### Clinical characteristics

Age, gender, body mass index (BMI), ASA grade, history of smoking and drinking, preoperative comorbidities (e.g. Hypertension, Diabetes, Coronary Heart Disease, etc.), presurgical pain score, surgical types (Laparoscopic radical surgery for gastric, colon, rectal and sigmoid cancer), duration of surgery and anesthesia, minimum body temperature, maximum partial pressure of end-respiratory carbon dioxide (PetCO_2_), estimated blood loss volume, urine output, and liquid intake. Moreover, presurgical pain was measured by using the Visual Analogue Scale (VAS).

#### Presurgical laboratory indicators

Red blood cell count, blood platelet count, blood type (A, B, AB, and O), glomerular filtration rate (GFR), urea nitrogen, uric acid, creatinine, low-density lipoprotein (LDL), high-density lipoprotein (HDL), aspartate aminotransferase (AST), alanine aminotransferase (ALT), AST/ALT, sodium, potassium, calcium, chloride, white blood cell count, hemoglobin, fasting blood glucose (FBG), Alb, TC, NLR, and LMR. Besides, the AST/ALT was calculated as aspartate aminotransferase divided by alanine aminotransferase. The NLR referred to neutrophil count divided by lymphocyte count, and the LMR was computed as lymphocyte count divided by monocyte count.

#### Postoperative outcomes

Use of postoperative analgesics, duration of postoperative hospitalization, length of ICU admission, and postoperative complications (e.g. infection, pain, postoperative nausea and vomiting, bleeding, anastomotic leakage, etc.).

### Naples prognostic score and postoperative delirium

#### Calculation of the NPS

**Fig. 1:**
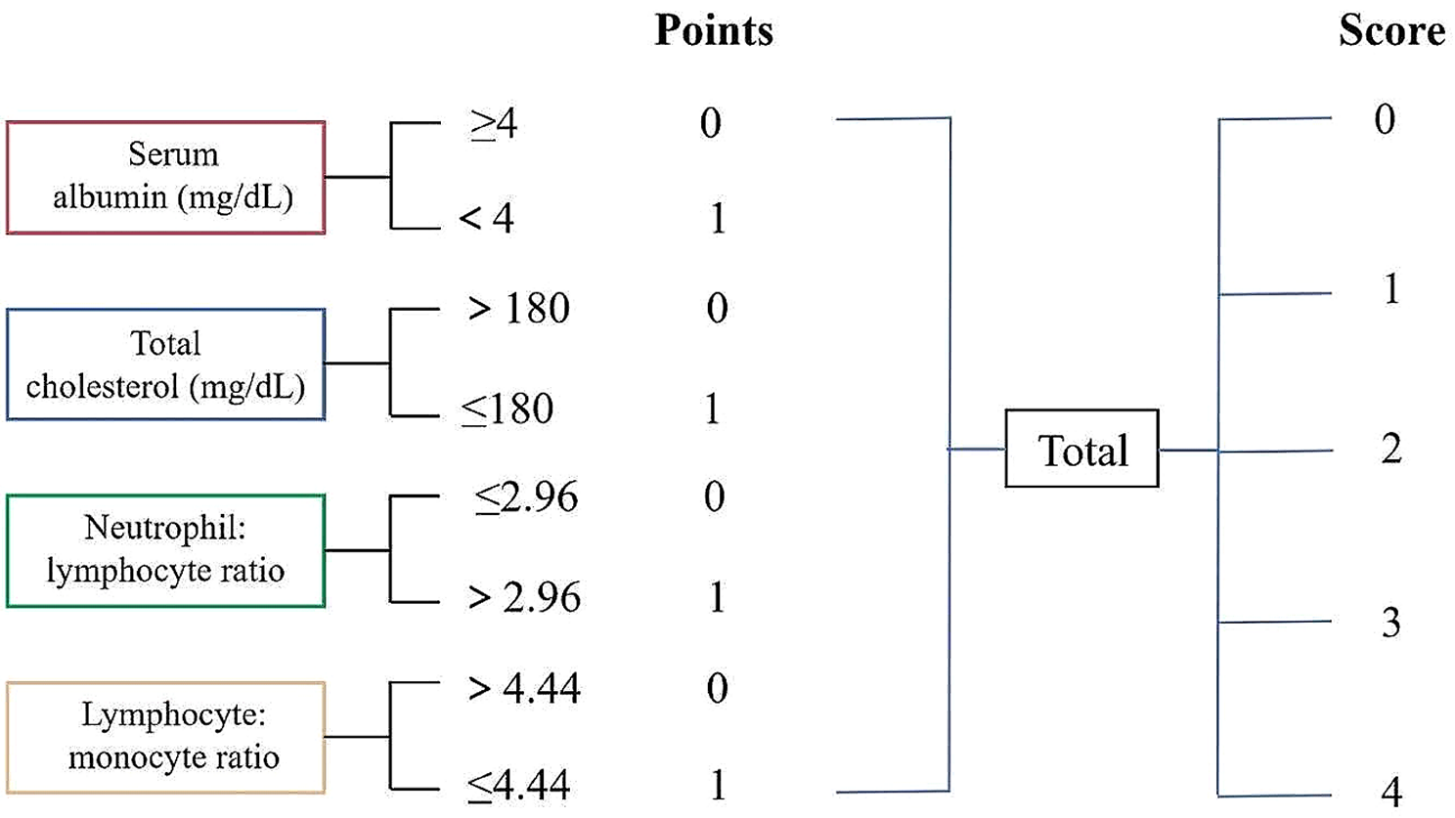
Calculation of the Naples prognostic score

As recently documented in this article [16], the NPS was computed from four parameters: serum Alb concentration, TC concentration, NLR, and LMR. Of these, serum Alb concentration < 4 g/dL was assigned 1 point and ≥ 4 g/dL was defined as 0 point; TC concentration < 180 mg/dL was scored as 1 point and ≥ 180 mg/dL was classified as 0 point; NLR ≥ 2.96 was defined as 1 point and < 2.96 was scored as 0 point; LMR < 4.44 was assigned 1 point and ≥ 4.44 was classified as 0 point. The NPS was composed of the sum of the above counts, as detailed in Fig. 1.

#### Delirium assessment

Firstly, we employed a chart-based approach to retrospectively define POD from the end of the procedure to before discharge [17]. Simultaneously, we examined in detail the complete electronic medical records and associated paper-based care reports for each enrolled patient to assess POD. Afterward, based on the above and the description of Xu et al [18], we finally determined the evaluation criteria of delirium: (i) when the patient was diagnosed with delirium by a psychiatrist; (ii) when the clinician prescribed antipsychotic drugs (e.g. quetiapine, olanzapine, and haloperidol) at any postoperative period; (iii) the diagnosis of delirium was agreed upon when two anesthesiologists performed a systematic screening of each enrolled patient. If decisions diverge, a third evaluator should be consulted to agree on a diagnosis of delirium).

### Estimated sample size

Initially, we retrospectively collected patient data from August 2023 to September 2023, and found that POD occurred in 4 out of 32 patients (12.5%). Moreover, recent literature indicated that the occurrence of POD in the elderly with gastrointestinal cancer ranged from 10.9–14.0% [19]. Hence, we stipulated that the incidence of POD in elderly gastrointestinal tumors was 12%, and estimated the required minimum sample size was 143 by using the G*Power Software, version 3.1 (with an alpha of 5%, the power of 80%, and the effect size of 70%) [20].

### Statistical analysis

All data were analyzed by SPSS 25.0 statistical software. Measurement data conforming to a normal distribution was presented as mean ± standard deviation (x ± s), and between-group comparisons were performed by independent sample t-test. Measurement data suitable for a skewed distribution were presented as median (interquartile range) [M(Q1-Q3)], and the Mann-Whitney U non-parametric test was applied for comparison between groups. Count data were presented as frequency (percent, %), and the comparison between groups was performed by Χ^2^ test or Fisher exact probability method. Multicollinearity between related variables was explained by tolerance (Tol) or variance inflation factor (VIF), where Tol < 0.01 or VIF > 10 indicated no collinearity between variables. We performed univariate analysis one by one for variables with statistical significance differences in baseline values, and then the indicators with statistically significant in univariate analysis were included in the multivariate logistic regression analysis. Next, the independent risk factors of POD in elderly patients with gastrointestinal cancer were screened, and the Hosmer-Lemeshow goodness-of-fit test was utilized to assess the fitting degree of the logistic multivariate model. Eventually, the predictive value of NPS to POD was conducted by the receiver operating characteristic (ROC) curve, and the area under the curve (AUC) was calculated. Among them, *p* < 0.05 for the differences were statistically significant.

## Results

### Characteristics of subjects

**Fig. 2:**
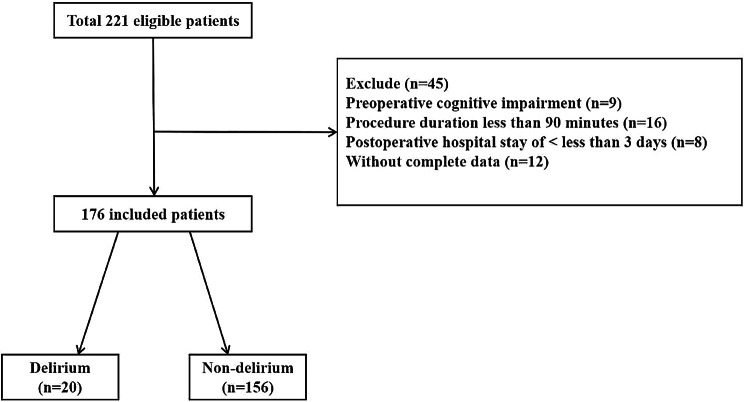
Flow chart of study population

Information from 221 subjects was initially collected for this study. After applying inclusion and exclusion criteria, we excluded 45 subjects, resulting in an enrollment of 176 patients in our analysis cohort. Our retrospective analysis revealed that delirium occurred in 20 patients (11.4%), which is consistent with the latest evidence [19]. A detailed screening flow chart was provided in Fig. 2, and the demographic characteristics of the subjects can be found in Table 1. Of the 176 patients, the overall mean age was 71 (65–76) and 96 (54.5%) subjects were aged ≥ 70 years, with 64 male and 112 female patients. The mean BMI was 24.38 ± 3.25 kg/m^2^. In addition, tumor location was found in 48 (27.3%) of both the stomach and rectum, 61 (34.7%) of the colon, and 17 (9.7%) of the sigmoid colon. In the baseline data, we found that subjects in the delirium group were significantly older than those in the non-delirium group [75(71.5–82.8) vs. 69(64.0–76.0), *P* < 0.001], and more subjects were aged ≥ 70[19(95.0) vs. 77(49.4), *P* < 0.001]. Besides, the delirium group had more patients with ASA grade ≥ 3 [18(90.0) vs. 88(56.4), *P* < 0.05]and higher preoperative pain scores [1(0–1) vs. 0(0–1), *P* < 0.05] than the non-delirium group.

**Table 1:** Comparison of demographic and clinical data between two groups

Variables		Overall (*n* = 176)	Delirium (*n* = 20)	Non-Delirium (*n* = 156)	*p*-value
Age (years)		71(65–76)	75(72–83)	69(64–76)	< 0.001*
Age ≥ 70, n (%)		96(54.5)	19(95.0)	77(49.4)	< 0.001*
Gender (male), n (%)		64(36.4)	6(30.0)	58(37.2)	0.530
BMI (kg/m^2^)		24.4 ± 3.2	24.2 ± 4.2	24.4 ± 3.1	0.799
ASA status, n (%)					0.004*
I/II		70(39.8)	2(10.0)	68(43.6)	
III/IV		106(60.2)	18(90.0)	88(56.4)	
Smoking history, n (%)		65(36.9)	7(35.0)	58(37.2)	0.849
Drinking history, n (%)		20(11.4)	4(20.0)	16(10.3)	0.196
Preoperative comorbidities, n (%)					
Hypertension		94(53.4)	14(70.0)	80(51.3)	0.114
Diabetes		43(24.4)	7(35.0)	36(23.1)	0.243
CHD		18(10.2)	4(20.0)	14(9.0)	0.126
Preoperative pain scores (VAS)		0(0–1)	1(0–1)	0(0–1)	0.002*
Surgical types, n (%)					0.794
Radical gastrectomy		48(27.3)	5(10.4)	43(27.6)	0.808
Colon cancer surgery		61(34.7)	7(35.0)	54(34.6)	0.973
Rectal cancer surgery		48(27.3)	7(35.0)	41(26.3)	0.410
Resection of sigmoid carcinoma		17(9.7)	1(5.0)	16(10.3)	0.449
Duration of surgery (min)		210(171–249)	217.5(152–267)	210(175–245)	0.812
Duration of anesthesia (min)		245(215–285)	265(216–294)	245(215–280)	0.425
Minimum body temperature (°C)		36.1(35.8–36.3)	36.2(35.7–36.3)	36.1(35.8–36.2)	0.407
Maximum PetCO_2_ (mmHg)		38(36–41)	39(36–42)	38(36–41)	0.463
Estimated blood loss volume (mL)		20(20–50)	20(20–30)	20(20–50)	0.379
Estimated urine volume (mL)		300(200–500)	300(200–400)	300(200–500)	0.217
Estimated liquid intake (mL)		2500(2000–3000)	2500(2125–3000)	2500(2000–3000)	0.532

*Notes* * *p* < 0.05 between delirium and non-delirium groups. *Abbreviations*: BMI, Body Mass Index; ASA, American Society of Anesthesiologists; CHD, Coronary Heart Disease; PetCO_2_, Partial Pressure of End-respiratory Carbon Dioxide

### Preoperative laboratory data

From preoperative laboratory data, it was observed that patients in the delirium group had lower preoperative GFRs [85.77(71.51–89.99) vs. 88.69(81.91–95.03), *P* < 0.05] and higher levels of creatinine [76.4(61.5–85.8) vs. 68.4(59.3–75.8), *P* < 0.05] than those in the non-delirium group. Additionally, the presurgical white blood cell count [7.14(6.04–8.34) vs. 5.79(4.64–7.23), *P* < 0.05] and FBG [6.37(5.80–7.49) vs. 5.40(4.90–6.40), *P* < 0.05] were obviously higher in the delirium group than in the non-delirium group. Most importantly, we explored that the subjects in the delirium group had higher preoperative NPS, and the difference was statistically significant between groups [3.5(3.0–4.0) vs. 2.0(1.0–3.0), *P* < 0.001]. Detailed laboratory indicators between the two groups were shown in Table 2.

**Table 2:** Preoperative laboratory variables in patients with or without POD

Variables	Overall (*n* = 176)	Delirium (*n* = 20)	Non-Delirium (*n* = 156)	*p*-value	
Red blood cell count (x10^12^/L)	4.1(3.6–4.5)	4.0(3.2–4.4)	4.1(3.6–4.6)	0.169	
Blood platelet count (x10^9^/L)	222(175–284)	228(174–343)	222(175–285)	0.837	
Blood type, n (%)					
A	48(27.3)	4(20.0)	44(28.2)	0.438	
B	65(36.9)	6(30.0)	59(37.8)	0.495	
AB	17(9.7)	4(20.0)	13(8.3)	0.109	
O	46(26.1)	6(30.0)	40(25.6)	0.676	
GFRs (ml/min)	88.2(81.3–94.7)	85.8(71.5–89.9)	88.7(81.9–95.0)	0.026*	
Urea nitrogen (mmol/L)	5.0(4.3–6.2)	5.6(3.7-7.0)	4.9(4.3–6.2)	0.395	
Uric acid (µmol/L)	280.1(238.9-329.1)	282.4(229.3-345.9)	280.1(238.9-329.1)	0.918	
Creatinine (µmol/L)	68.9(59.9–76.4)	76.4(61.5–85.8)	68.4(59.3–75.8)	0.028*	
LDL (mmol/L)	2.9 ± 0.9	2.6 ± 0.8	2.9 ± 0.9	0.082	
HDL (mmol/L)	1.1(0.9–1.3)	1.0(0.9–1.3)	1.1(0.9–1.4)	0.180	
Triglyceride (mmol/L)	1.1(0.9–1.5)	1.1(0.8–1.4)	1.1(0.9–1.5)	0.767	
AST (U/L)	18.8(16.1–23.4)	19.3(16.7–22.4)	18.8(16.1–23.5)	0.785	
AST/ALT	1.5(1.2–1.8)	1.6(1.3–1.9)	1.5(1.2–1.8)	0.538	
Sodium (mmol/L)	140(138–141)	141(138–142)	140(138–141)	0.190	
Potassium (mmol/L)	4.0 ± 0.4	3.9 ± 0.5	4.0 ± 0.4	0.394	
Calcium (mmol/L)	2.3(2.2–2.4)	2.3(2.2–2.3)	2.3(2.2–2.4)	0.309	
Chloride (mmol/L)	106(103–107)	106(104–109)	106(103–107)	0.294	
White blood cell (x10^9^/L)	6.0(4.7–7.4)	7.1(6.0-8.3)	5.8(4.6–7.2)	0.008*	
Hemoglobin (g/L)	116.8 ± 21.7	116.6 ± 20.3	116.9 ± 21.9	0.952	
FBG (mmol/L)	5.5(4.9–6.4)	6.4(5.8–7.5)	5.4(4.9–6.4)	0.008*	
Serum album (g/L)	38.8 ± 4.0	36.8 ± 4.3	39.0 ± 3.9	0.021*	
Total cholesterol (mmol/L)	173.8 ± 40.8	156.6 ± 35.0	175.9 ± 41.0	0.045*	
NLR	2.7(2.0-3.8)	3.4(3.0-4.2)	2.7(1.8–3.6)	0.007*	
LMR	4.4(3.1–5.9)	3.2(2.6–4.3)	4.5(3.4–5.9)	0.005*	
NPS	2.0(1.0–3.0)	3.5(3.0–4.0)	2.0(1.0–3.0)	< 0.001*	

*Notes* * *p* < 0.05 between delirium and non-delirium groups. *Abbreviations* POD, Postoperative Delirium; GFRs, Glomerular Filtration Rates; LDL, Low-density Lipoprotein; HDL, High-density Lipoprotein; AST, Aspartate aminotransferase; ALT, Alanine aminotransferase; FBG, Fasting Blood Glucose; NLR, Neutrophil-to-Lymphocyte Ratio; LMR, Lymphocyte-to-monocyte Ratio; NPS, Naples Prognostic Score

### Multicollinearity analysis of related risk factors for POD

We implemented a multicollinearity diagnosis of related risk factors with POD, and the complete data were recorded in Supplementary Table 1, which illustrated that there was no multiple linear relationship between these potential risk factors.

### Independent risk factors for POD

Initially, we detected that NPS, age, ASA grade ≥ 3, creatinine, GFRs, white blood cell count, and FBG were identified as risk factors for POD through univariate logistic regression analysis in unadjusted results. Finally, our multivariate logistic regression analysis demonstrated that independent risk factors related to POD included NPS (OR = 2.956, 95% CI = 1.556–5.618, *P* = 0.001), age (OR = 1.097, 95% CI = 0.997–1.207, *P* = 0.049), ASA grade ≥ 3 (OR = 8.402, 95% CI = 1.102–64.051, *P* = 0.04), and FBG (OR = 1.837, 95% CI = 1.193–2.939, *P* = 0.006). What’s more, the Hosmer-Lemeshow goodness-of-fit test pointed out the multiple regression model fitted very well with a χ^2^ value of 4.172 and *P*-value of 0.841. And the remaining details were presented in Table 3.

**Table 3:** Analyses of risk factors for POD

Variables	Univariate			Multivariate		
	OR	95% CI	*p*-value	OR	95% CI	*p*-value
Age (years)	1.144	1.062–1.232	< 0.001*	1.097	0.997–1.207	0.049*
ASA status ≥ 3	6.955	1.560-31.005	0.011*	8.402	1.102–64.051	0.040*
Preoperative pain scores	2.217	0.810–6.065	0.121	--	--	--
Creatinine (µmol/L)	1.030	1.003–1.058	0.028*	--	--	--
GFRs (ml/min)	0.983	0.961–1.006	0.140	--	--	--
White blood cell count (x10^9^/L)	1.299	1.041–1.622	0.021*	--	--	--
FBG (mmol/L)	1.457	1.076–1.974	0.015*	1.837	1.193–2.939	0.006*
NPS	3.061	1.764–5.312	< 0.001*	2.956	1.556–5.618	0.001*

*Notes*: * *p* < 0.05 between delirium and non-delirium groups. *Abbreviations*: POD, Postoperative Delirium; ASA, American Society of Anaesthesiologists; CI, Confidence Interval; OR, Odds Ratio; GFRs, Glomerular Filtration Rates; FBG, Fasting Blood Glucose; NPS, Naples Prognostic Score

### Predictive value of NPS for POD

**Fig. 3:**
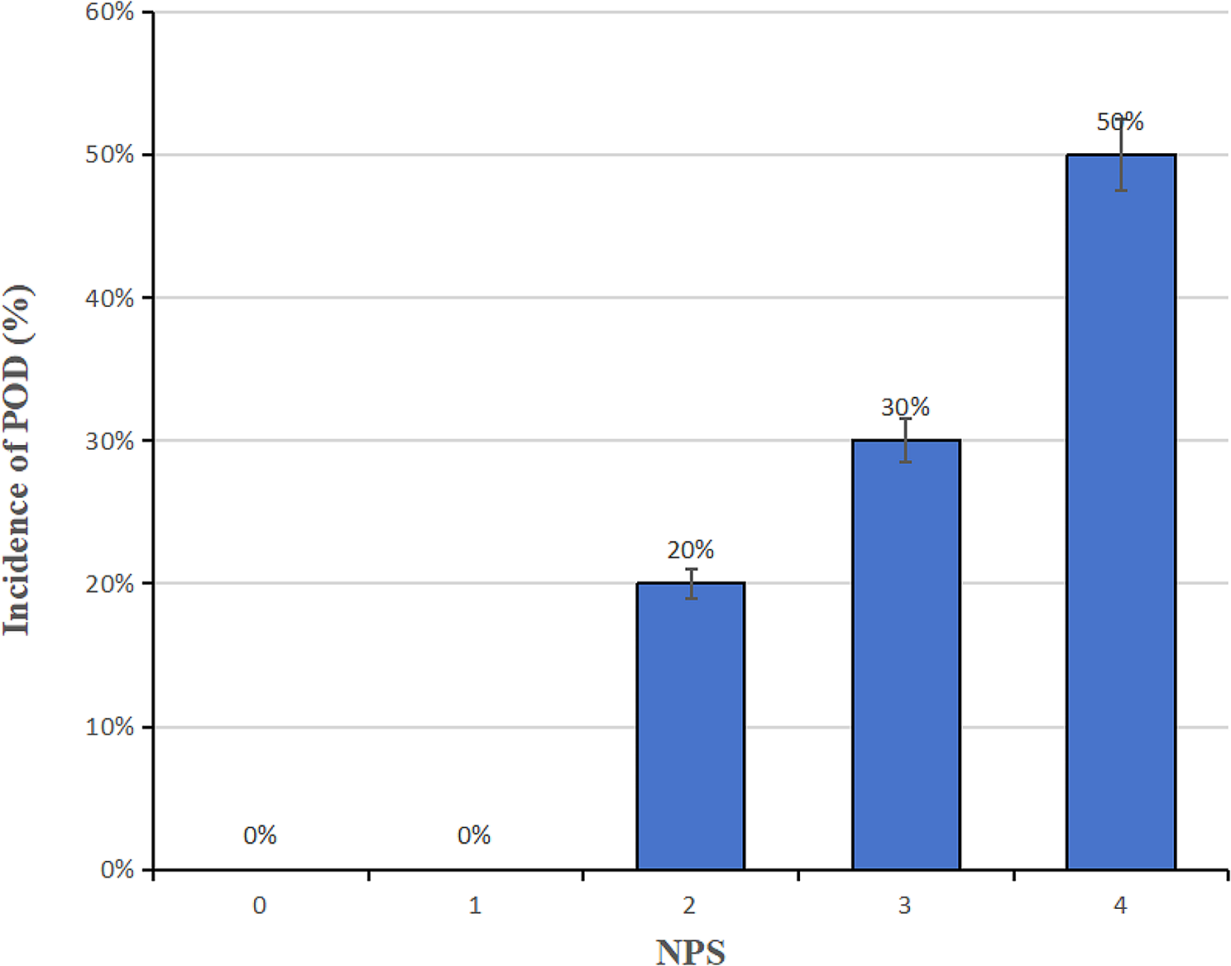
Incidence of POD in various NPS. *Abbreviation* POD, Postoperative Delirium; NPS, Naples Prognostic Score.

**Fig. 4:**
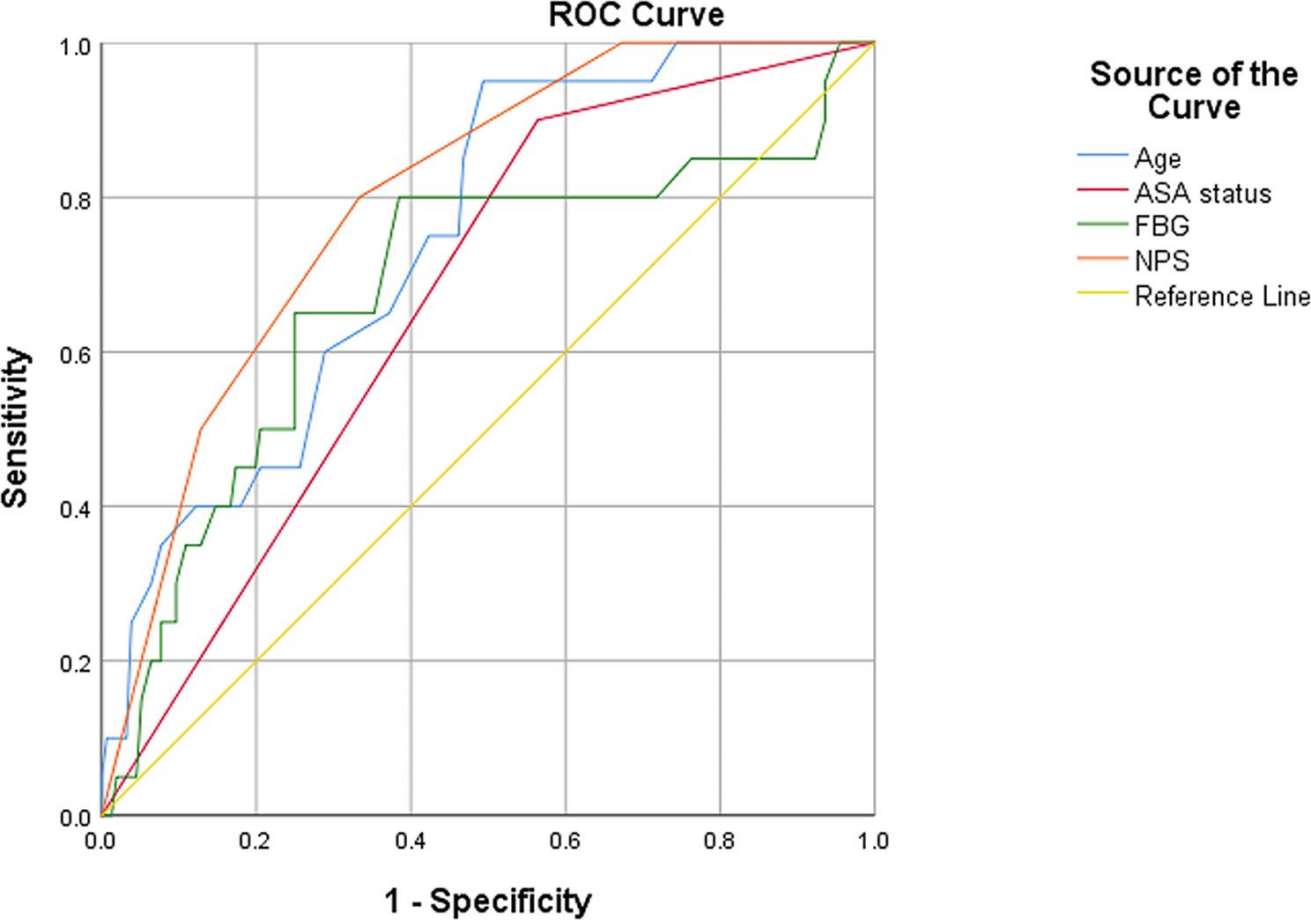
The predictive value of NPS for POD by ROC analysis. *Abbreviation* NPS, Naples Prognostic Score; POD, Postoperative Delirium; ROC, Receiver Operating Characteristic; AUC, Area Under the Curve.

According to the NPS calculation process, we could divide it into five values (0–4), and interestingly, among all patients who developed a POD, the 4-point was 50%, the 3-point was 30%, and the 2-point was only 20%, and 0-point or 1-point didn’t occur. The full results were shown in Fig. 3. Moreover, the ROC was utilized to further assess the value of the detected independent risk factors in predicting POD, and the results were demonstrated in Table 4. From the data table, it could be concluded that the NPS had the largest AUC with 0.798 and sensitivity of 0.800, compared to other risk factors (age AUC: 0.746, sensitivity: 0.600, *p*<0.001; ASA status ≥ 3 AUC: 0.668, sensitivity: 0.900, *p* = 0.015; FBG AUC: 0.682, sensitivity: 0.750, *p* = 0.008) for POD. Further, based on the ROC analysis, we calculated 2.5 as the optimal cut-off for NPS for POD prediction accuracy, and detailed data were recorded in Fig. 4.

**Table 4:** The accuracy of risk factors to predict POD by ROC curve analysis

Variables	Area under the curve (95% confidence interval)	Sensitivity	Specificity	Cut-off value	*P*-value	
Age (years)	0.746(0.646–0.846)	0.600	0.712	74.50	< 0.001	
ASA status ≥ 3	0.668(0.560–0.776)	0.900	0.436	1.50	0.015	
FBG (mmol/L)	0.682(0.555–0.809)	0.750	0.564	6.10	0.008	
NPS	0.798(0.710–0.886)	0.800	0.667	2.50	< 0.001	

*Abbreviations*: POD, Postoperative Delirium; ROC, Receiver Operator Characteristic; FBG, Fasting Blood Glucose; NPS, Naples Prognostic Score

### Outcomes of patients

As shown in Table 5, we retrospectively explored clinical outcomes in both groups of patients. We discovered that the composite rate of postoperative complications was significantly higher in the delirium group than in the non-delirium group [20(100) vs. 89(57.1), *P* < 0.001], but was not found in a single prevalence of postoperative complications. Additionally, no significant discrepancy was detected in the use of postoperative analgesics, length of hospitalization, and ICU stay between the two groups.

**Table 5:** Comparison of clinical outcomes in both groups

Outcomes	Overall (*n* = 176)	Delirium (*n* = 20)	Non-Delirium (*n* = 156)	*p*-value
Use of postoperative analgesics, n (%)	154(87.5)	16(80.0)	138(88.5)	0.281
Duration of postoperative hospitalization (d)	10(8–12)	11(9–17)	9(8–12)	0.070*
Length of ICU stay (d)	0(0–0)	0(0–0)	0(0–0)	0.151
Postoperative complications, n (%)	109(61.9)	20(100.0)	89(57.1)	< 0.001*
Postoperative infection	77(43.8)	10(50.0)	67(42.9)	0.550
Postoperative pain	38(21.6)	5(25.0)	33(21.2)	0.773
Postoperative nausea and vomiting	8(4.5)	1(5.0)	7(4.5)	1.000
Postoperative bleeding	3(1.7)	0(0.0)	3(1.9)	1.000
Anastomotic leakage	1(0.6)	0(0.0)	1(0.6)	1.000

*Notes* * *p* < 0.05 between delirium and non-delirium groups. **Abbreviations**: ICU, Intensive Care Unit

## Discussion

POD, a common and severe issue in the current context, takes place in 10.9–14.0% of older adults after gastrointestinal tumor surgery, which slows the recovery of body functions to baseline levels and leads to augmented hospital length of stay and expenditures [21, 22]. Fortunately, POD could be prevented, and exploring potential risk factors for delirium and early intervention were the first step in treating POD [23]. Over the past few years, several studies had examined various independent risk factors associated with POD in patients underwent gastrointestinal tumor surgery [21, 24]. Notably, our current study investigated the predictive power of preoperative NPS for POD in patients underwent gastrointestinal tumor surgery. To the best of our knowledge, this was the first clinical study to predict POD using a composite indicator of NPS. Our analysis confirmed that preoperative NPS might be an effective predictor of POD in the elderly underwent gastrointestinal tumor surgery.

The present experiment unveiled a POD incidence of 11.4%, which was in line with the previous information of POD in the elderly underwent surgery for gastrointestinal tumors [3]. However, other articles indicated the occurrence of POD was a little higher than that in our trial, such as the 27.4% by Chen et al. and 26.1% by Choi et al., which might be relevant to type of operation, sample size and baseline characteristics in patients [25, 26]. Also, we found a higher incidence of other postoperative complications in the delirium group, which was consistent with previous literature [27]. Similarly, another article suggested that POD was a potential risk factor for comorbidities and adverse outcomes following total hip and knee arthroplasty [28]. Consequently, it is evidence that POD might directly or indirectly affect the occurrence of postoperative adverse outcomes, so clinicians and anesthesiologists should evaluate delirium as early as possible after surgery in order to effectively prevent or mitigate POD and its associated complications.

In view of this, the identification of risk factors correlated with delirium was considered as an essential part in high-risk populations of the POD [29, 30]. Our univariate logistic regression analysis confirmed that 6 data were potential risk factors of POD, including age, ASA grade (≥ 3), among others. After adjusting for confounding factors by multivariate logistic regression analysis, age, ASA grade ≥ 3, FBG and NPS were considered as an independent predictor of POD. It was well known that advanced age was a potential risk factor of POD in vast majority of patients underwent surgery [31]. Surgical patients aged 65 years and older had unresolved high-risk factors, as the aging brain was more vulnerable to peripheral inflammation and immune response of body [32]. At the same time, our results suggested that the high ASA grade (≥ 3) might increase the occurrence of POD in the elderly with gastrointestinal tumors. Having a high ASA grade implied that the patients had severe systemic disease, functional impairment along with great risk of anesthesia [33]. Although no significant differences were detected in our trial between groups in preoperative comorbidities (e.g. Hypertension, Diabetes, CHD, etc.), we could not deny the detrimental effect of preoperative physical status on POD. Additionally, we detected that FBG was an independent risk factor for POD development in the elderly with gastrointestinal tumors, consistent with the results of Liu et al., who suggested that elevated preoperative FBG significantly increased POD by T-tau in CSF [34].

More importantly, we explored that NPS was an independent risk factor of POD in our trail. The NPS primarily comprised total cholesterol concentration, Alb concentration, NLR and LMR. Previous studies showed that preoperative markers of nutritional-immune status, such as blood lipids, HDL, Alb and white blood cell count, were regarded an independent risk factor of POD in the elderly underwent surgery [35–37]. Hypoalbuminemia was a marker not only of malnutrition but also of systemic inflammation. Unfortunately, a few pro-inflammatory mediators, such as cytokines and complement proteins, could affect albumin concentrations [38]. Total cholesterol concentration changed easily in hospitalized patients due to variations in body fluid levels [39]. Similarly, a previous study demonstrated that numerous biomarkers in the inflammatory response were risk factors for POD, including c-reactive protein (CRP), interleukin-1 (IL-1), interleukin-6 (IL-6), and tumor necrosis factor (TNF-α) [40]. However, their clinical application was limited due to the expensiveness of taking blood samples. In most surgical patients, blood routine test must be measured preoperatively and are readily available [41]. In a word, a single index could be influenced by the host’s situation and even be misleading when there is an interaction between inflammatory factors and nutritional indicators. Therefore, the NPS was considered as a more valuable scoring system, which contained more blood parameters and was strongly associated with inflammation and nutritional-immune status.

In recent years, some articles had confirmed the connection between preoperative NPS and short-term and long-term complications in the elderly with GC and CRC [9, 42]. Also, a recent review noted that POD was the most common serious postoperative complication in the older individuals, with uncertain aetiology, limited preventative strategies, and poor long-term outcomes [43]. Whereas incidences and risk factors of POD were well reported, less was known about the correlation between NPS and POD after gastrointestinal tumor surgery. Our article firstly focused on the predictive role of NPS for POD in the elderly underwent gastrointestinal tumor surgery. It is evident from our results that preoperative NPS provided the greatest value for predicting POD, with an AUC of 0.798. Additionally, large number of articles showed that elderly cancer patients with high NPS had reduced recurrence-free survival (RFS) and overall survival (OS) [9, 13]. Interestingly, Elsa et al. testified that POD was associated with reduced RFS and OS within 5 years [44]. Therefore, further investigation into the specific association between NPS and POD was warranted to enhance post-surgery patient outcomes. Additionally, while the optimal cut-off value of NPS had been extensively studied in the context of cancer survival [14, 45]. Its exploration in predicting POD following gastrointestinal tumor surgery had been limited. In our study, we utilized ROC analysis to establish the optimal NPS threshold as 2.5, and found that NPS was a superior predictor of POD compared to other related variables. Consequently, the preoperative assessment of NPS holds promise in facilitating the early detection of POD and ameliorating patient outcomes following gastrointestinal tumor surgery.

Nevertheless, there remain some deficiencies in our experiment that need to be further optimized. First of all, this was a single-center, retrospective article, and we should pay more attention to explore the association between NPS and POD in future prospective trials. Secondly, our focus was solely on laparoscopic surgery, and the predictive value of preoperative NPS on POD for open surgery and other surgical types still requires further exploration. Thirdly, the lack of data on the status of chemoradiation before and after surgery in subjects with gastrointestinal tumors was a notable limitation. This missing information could potentially impact the patient’s preoperative nutritional status and oxidative stress, thereby disrupting the accurate calculation of the NPS. Finally, the nature of retrospective studies made it difficult to comprehensively evaluate preoperative physical, psychological, or cognitive function, which were potential confounding factors affecting the results, so more prospective trails were needed to validate our conclusions in the future. All in all, this was the first paper to examine the predictive value of preoperative NPS for POD in the elderly underwent gastrointestinal tumor surgery, but trial results should be viewed rationally.

## Conclusion

In this study, age, ASA grade ≥ 3, FBG, and NPS were identified as independent risk factors for POD in elderly patients who underwent gastrointestinal tumor surgery. Specifically, we found that preoperative NPS could more effectively predict the prevalence of POD.

### Electronic supplementary material

Below is the link to the electronic supplementary material.

Supplementary Material 1

## Data Availability

The datasets used and analyzed during the current study are available from the corresponding author on reasonable request.

## Abbreviations

POD: Postoperative delirium
NPS: Naples prognostic score
ROC: Receiver operating characteristic curves
AUC: The area under the curve
GC: Gastric cancer
CC: Colon cancer
CSF: Cerebrospinal fluid
MLR: Monocyte-to-lymphocyte ratio
ICU: Intensive care unit
Alb: Albumin
TC: Total cholesterol
NLR: neutrophil-to-lymphocyte ratio
ASA: American Society of Anesthesiologists
RC: Rectal cancer
BMI: Body mass index
VAS: Visual analogue scale
GFR: Glomerular filtration rate
LDL: Low-density lipoprotein
HDL: High-density lipoprotein
AST: Aspartate aminotransferase
ALT: Alanine aminotransferase
FBG: Fasting blood glucose
Tol: Tolerance
VIF: Variance inflation factor
CRP: C-reactive protein
IL-1: Interleukin-1
IL-6: Interleukin-6
TNF-α: Tumor necrosis factor
RFS: Recurrence-free survival
OS: Overall survival
