# Supplementary material for: Predictive value of the Naples prognostic score on postoperative delirium in the elderly with gastrointestinal tumors: a retrospective cohort study
Source: BMC Geriatr. 2024 Jun 20;24:535. doi: 10.1186/s12877-024-05113-y (PMC11188257; doi:10.1186/s12877-024-05113-y)
Supplement: Supplementary file 1 — Supplementary Material 1 [file 12877_2024_5113_MOESM1_ESM.docx]

| **Additional file 1 Multicollinearity analysis of related risk factors for POD** | | |
| --- | --- | --- |
| **Variables** | **Tolerance** | **VIF** |
| Age (years) | 0.738 | 1.356 |
| ASA status≥3 | 0.741 | 1.350 |
| Creatinine (μmol/L) | 0.952 | 1.050 |
| White blood cell (x10^9^/L) | 0.968 | 1.033 |
| FBG (mmol/L) | 0.963 | 1.038 |
| NPS | 0.936 | 1.069 |
| **Notes:** Tolerance＞0.01 or VIF＜10 means no collinearity.  **Abbreviations:** POD, Postoperative Delirium; ASA, American Society of Anaesthesiologists; VIF, Variance Inflation Factor; GFR, Glomerular Filtration Rate; NPS, Naples Prognostic Score. | | |
